# Patterns of gene flow and selection across multiple species of *Acrocephalus* warblers: footprints of parallel selection on the Z chromosome

**DOI:** 10.1186/s12862-016-0692-2

**Published:** 2016-06-16

**Authors:** Radka Reifová, Veronika Majerová, Jiří Reif, Markus Ahola, Antero Lindholm, Petr Procházka

**Affiliations:** Department of Zoology, Faculty of Science, Charles University in Prague, Prague, Czech Republic; Institute for Environmental Studies, Faculty of Science, Charles University in Prague, Prague, Czech Republic; Department of Zoology and Laboratory of Ornithology, Faculty of Science, Palacký University in Olomouc, Olomouc, Czech Republic; Department of Biology, Section of Ecology, FI-20014 University of Turku, Turku, Finland; Natural Resources Institute Finland, Itäinen Pitkäkatu 3, FI-20240 Turku, Finland; Tornfalksvägen 2 bst 15, Esbo, Finland; Institute of Vertebrate Biology, Academy of Sciences of the Czech Republic, Brno, Czech Republic

**Keywords:** Adaptive radiation, Speciation, Gene flow, Parallel adaptive evolution, Z chromosome, *Acrocephalus* warblers

## Abstract

**Background:**

Understanding the mechanisms and selective forces leading to adaptive radiations and origin of biodiversity is a major goal of evolutionary biology. *Acrocephalus* warblers are small passerines that underwent an adaptive radiation in the last approximately 10 million years that gave rise to 37 extant species, many of which still hybridize in nature. *Acrocephalus* warblers have served as model organisms for a wide variety of ecological and behavioral studies, yet our knowledge of mechanisms and selective forces driving their radiation is limited. Here we studied patterns of interspecific gene flow and selection across three European *Acrocephalus* warblers to get a first insight into mechanisms of radiation of this avian group.

**Results:**

We analyzed nucleotide variation at eight nuclear loci in three hybridizing *Acrocephalus* species with overlapping breeding ranges in Europe. Using an isolation-with-migration model for multiple populations, we found evidence for unidirectional gene flow from *A. scirpaceu*s to *A. palustris* and from *A. palustris* to *A. dumetorum*. Gene flow was higher between genetically more closely related *A. scirpaceus* and *A. palustris* than between ecologically more similar *A. palustris* and *A. dumetorum*, suggesting that gradual accumulation of intrinsic barriers rather than divergent ecological selection are more efficient in restricting interspecific gene flow in *Acrocephalus* warblers. Although levels of genetic differentiation between different species pairs were in general not correlated, we found signatures of apparently independent instances of positive selection at the same two Z-linked loci in multiple species.

**Conclusions:**

Our study brings the first evidence that gene flow occurred during *Acrocephalus* radiation and not only between sister species. Interspecific gene flow could thus be an important source of genetic variation in individual *Acrocephalus* species and could have accelerated adaptive evolution and speciation rate in this avian group by creating novel genetic combinations and new phenotypes. Independent instances of positive selection at the same loci in multiple species indicate an interesting possibility that the same loci might have contributed to reproductive isolation in several speciation events.

**Electronic supplementary material:**

The online version of this article (doi:10.1186/s12862-016-0692-2) contains supplementary material, which is available to authorized users.

## Background

Interspecific gene flow is an important evolutionary force. It may enrich genetic variation of individual species, facilitate the origin of new phenotypes, lead to the origin of new species, or, conversely, to species fusion [1]. It has been suggested that high speciation rates and rapid phenotypic changes observed during adaptive radiations may be facilitated by increased genetic variation and novel genetic combinations produced by introgressive hybridization among the species [2, 3]. Patterns of interspecific gene flow can also provide an important insight into the genetic basis of reproductive isolation [4]. In species with incomplete reproductive isolation, gene flow occurs at loci with alleles that are neutral or beneficial on the genomic background of the other species, but is limited at loci harboring alleles involved in reproductive isolation [5].

Most work exploring patterns and rates of interspecific gene flow focused on pairs of closely related sister species [6] because, until recently [7], models allowing the analysis of interspecific gene flow were limited to only two species. Several general findings emerged from these studies. First, in the case of speciation driven by intrinsic barriers, interspecific gene flow is often reduced on the sex chromosomes (i.e., XY in heterogametic male organisms and ZW in heterogametic female organisms) [8–11] and in genomic regions with low recombination rates [12], indicating that genetic incompatibilities tend to preferentially accumulate in these genomic regions. Second, in the case of speciation driven by divergent ecological selection, gene flow is correlated with ecological divergence [13] and is reduced around genes underlying ecological differences [14]. Interspecific gene flow across larger sections of the phylogenetic tree is, however, much less studied. Such studies would be particularly interesting in instances of rapid radiations, where lineage divergence occurs on a shorter timescale than the completion of reproductive isolation [15–18]. Knowledge of patterns and rates of interspecific gene flow in such systems allows to address questions, which are difficult or even impossible to study using models of only two sister species. One can, for example, examine whether the same genes contribute to reproductive isolation in different speciation events, assess the importance of various reproductive barriers in restricting the gene flow and determine the order in which different reproductive barriers arise during lineage divergence. This information can help us to understand why lineage divergence occurs so quickly in some taxonomic groups, while not in others.

*Acrocephalus* warblers are small passerines that underwent an adaptive radiation during the last approximately 10 million years [19], which gave rise to 37 extant species occupying mainly Eurasia, Africa, Australia and Pacific islands [20]. The members of this genus are phenotypically quite uniform. They, however, display a great diversity in ecology and behaviour, and have been used extensively as models in ecological and behavioral research [20]. Interspecific hybridization occasionally occurs in *Acrocephalus* warblers. It has been reported not only between sister species (e.g., *A. scirpaceus* × *A. palustris*, *A. arundinaceus* × *A. stentoreus*), but also between more distantly related species (e.g., *A. palustris* × *A. dumetorum*, *A. palustris* × *A. schoenobaenus*, *A. scirpaceus* × *A. arundinaceus*) (see Additional file 1 for all reported cases of interspecific hybridization in *Acrocephalus* warblers and references). In some of these cases, hybridization occurs between species of very different body size (*A. scirpaceus* × *A. arundinaceus*) or even between species belonging to different subgenera (e.g. *A. palustris* × *A. schoenobaenus*). So far very little is known about the degree of postzygotic reproductive isolation between the species. In some cases, e.g. between sister species *A. arundinaceus* and *A. stentoreus*, postzygotic isolation seems to be quite strong as no backcross hybrids have been observed in a hybridizing population [21]. In other cases (e.g. between *A. palustris* and *A. dumetorum*), however, observation of fertile F_1_ hybrids [22] suggests that postzygotic isolation is incomplete and interspecific gene flow might occur between the species.

Here we performed the first study exploring patterns and rates of interspecific gene flow in this avian group, focusing on three species of the subgenus *Notiocichla*: *A. scirpaceus*, *A. palustris* and *A. dumetorum*. All three species are very similar in morphology, but have different habitat requirements, migration strategies and song patterns [23]. *A. scirpaceus* breeds across Europe, northern Africa and western Asia typically in reed beds and winters in sub-Saharan Africa. Its sister species, *A. palustris*, has similar geographical distribution, but occupies mostly damp herbaceous vegetation and winters in southeastern Africa. Males of each species sing different songs, nevertheless, in *A. palustris*, mixed singers that incorporate song phrases from *A. scirpaceus* to its repertoire are known [24]. Both species occasionally form mixed pairs [24] and are able to produce viable hybrid offspring (e.g. [25]; see Additional file 1 for a complete list of references). *A. dumetorum*, the sister species to the *A. scirpaceus* and *A. palustris* clade [26], breeds in Asia and Northeastern Europe in various herbaceous or bushy vegetation and winters in India. In Eastern Europe and Western Asia, it often co-occurs with *A. palustris* at the same sites and hybridization between the two species has been observed (e.g. [22, 27]; see Additional file 1 for a complete list of references). The exact frequency of hybridization between *A. palustris* and *A. scirpaceus* and between *A. palustris* and *A. dumetorum* is hard to estimate owing to morphological similarity of the species and difficulty to distinguish hybrids without genetic analysis. The total number of observed mixed pairs is, however, comparable in both species pairs (Additional file 1). The cases of hybridization between *A. dumetorum* and *A. scirpaceus,* which are ecologically more divergent*,* are not known, although one putative hybrid individual has been reported [28].

To get the first insight into mechanisms and selective forces driving *Acrocephalus* radiation, we analyzed nucleotide variation at four autosomal and four loci on the Z chromosome in sympatric populations of *A. scirpaceus*, *A. palustris* and *A. dumetorum*. Using an isolation-with-migration model for multiple populations we tested whether interspecific gene flow occurred among the studied species and if so, whether it was higher between more genetically similar or between more ecologically similar species. In addition, using several tests of neutrality, we explored patterns of positive selection in all three species.

## Methods

### Study area and samples

**Fig. 1:**
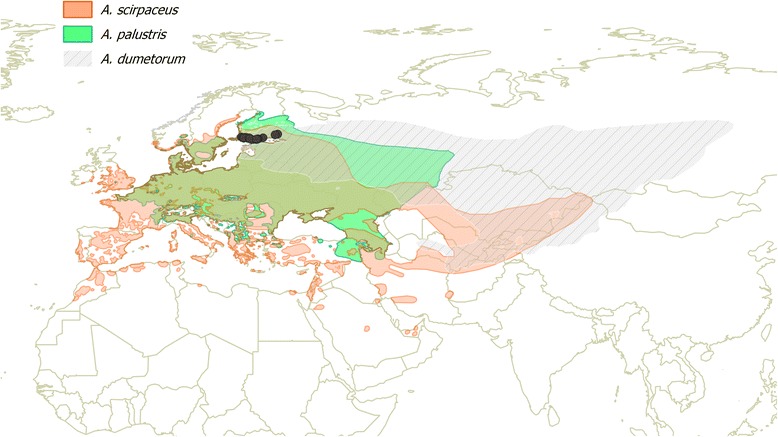
Breeding ranges of the three studied *Acrocephalus* warblers, *A. scirpaceus*, *A. palustris* and *A. dumetorum*. Sampling sites are indicated by dark grey circles

We analyzed samples from 22 individuals of *A. scirpaceus*, 26 individuals of *A. palustris* and 25 individuals of *A. dumetorum*. All birds were caught in 2010 during the breeding season (June and early July) in southern Finland, where the breeding ranges of all three species overlap (Fig. 1). Coordinates of the sites, where individual birds were captured, are shown in Additional file 2. From each individual, a blood sample was collected by brachial vein puncture and stored in pure ethanol for further extraction of genomic DNA. In addition, a sample from one individual of *A. schoenobaenus* was used as the outgroup.

### Molecular sex determination and sequencing

Genomic DNA was purified by DNeasy Tissue Kit (Qiagen) according to manufacturer’s instructions. Sex of individual birds was determined following the method of Griffiths et al. [29] using P2 and P8 primers. These primers amplify a fragment of two homologous genes located on the Z and W chromosomes. The amplified fragments from the Z and W chromosomes differ in length, which can be visualized on agarose gel. We further amplified and sequenced introns of four autosomal and four Z-linked loci. The four autosomal loci are located on different chromosomes in the Zebra Finch (*Taeniopygia guttata*) genome. Of the four Z-linked genes, *PPWD1* and *ADAMTS6* are close physically (68 kb), and therefore may not have independent evolutionary histories. However, the other two loci on the Z chromosome are separated from each other and from PPWD1 and ADAMTS6 by > 19 Mb. Primers for PCR amplification were partially obtained from published studies and partially designed by ourselves. All primers were designed in conserved exonic regions of the Zebra Finch or chicken (*Gallus gallus*) genome in order to amplify intronic sequences. Primer sequences and lengths of the obtained PCR products are provided in Additional file 3. PCR conditions are same as in Storchová et al. [9]. All PCR products were sequenced in both directions with the PCR primers using Sanger sequencing at the DNA sequencing laboratory at Charles University in Prague.

### Data analyses

Sequences were manually edited using CodonCode Aligner software (CodonCode Corporation, Dedham, MA). Alignments were generated by ClustalW as implemented in the program BioEdit [30]. All alignments were visually checked and manually adjusted. Exonic sequences as well as indel polymorphisms were excluded from the analyses. Individuals and/or positions with missing data (mostly at the ends of the sequences) were eliminated from the dataset to get sequences of the same length for each locus. Diploid sequences at each locus were further separated into two haplotypes using the program PHASE, version 2.1.1 [31] with the following parameters: number of iterations = 10,000, thinning interval = 1, burnin = 1000. We used the default recombination model, which is the general model for varying recombination rate. For each dataset, we applied the algorithm five times with different random seeds, and we checked for consistency of results across independent runs. We obtained identical haplotypes across all runs for all loci. The final number of haploid sequences for each locus and the length of sequences are shown in Table 1.

**Table 1:** Polymorphism statistics for eight studied loci

Locus	Chr^a^	L^b^	Species^c^	N^d^	S^e^	π (%)^f^	θ (%)^g^	TD^h^	FLD^i^	D (%)^j^
*17483*	A	484	*A.s.*	44	9	0.323	0.427	−0.701	−0.566	3.273
			*A.p.*	32	13	0.805	0.667	0.675	0.149	3.648
			*A.d.*	48	10	0.268	0.466	−1.225	−1.641	3.594
			All	124	32	0.731	1.226			3.494
*21281*	A	355	*A.s.*	28	29	1.868	2.099	−0.403	−1.197	2.998
			*A.p.*	20	24	1.893	1.906	−0.025	−0.200	2.634
			*A.d.*	38	22	1.071	1.475	−0.922	−0.823	3.136
			All	86	50	1.905	2.802			2.974
*24972*	A	636	*A.s.*	24	29	0.999	1.221	−0.683	−1.224	2.732
			*A.p.*	38	35	0.819	1.310	−1.314	−2.431	2.557
			*A.d.*	38	30	0.861	1.123	−0.809	−0.404	2.578
			All	100	71	1.031	2.156			2.607
*RPL5-4*	A	390	*A.s.*	38	4	0.101	0.244	−1.420	−2.135	3.893
			*A.p.*	50	15	0.994	0.859	0.484	1.112	4.359
			*A.d.*	48	12	0.591	0.693	−0.438	0.407	4.049
			All	136	35	1.383	1.636			4.120
*ADAMTS6*	Z	488	*A.s.*	35	0	0.000	0.000	/	/	1.025
			*A.p.*	43	2	0.019	0.095	−1.480	−2.462	0.829
			*A.d.*	42	7	0.206	0.333	−1.050	−1.002	0.937
			All	120	10	0.171	0.382			0.924
*PPWD1*	Z	566	*A.s.*	34	14	0.550	0.605	−0.295	0.228	2.520
			*A.p.*	45	9	0.318	0.364	−0.358	0.712	2.493
			*A.d.*	13	15	0.716	0.854	−0.676	−0.795	2.202
			All	92	39	0.663	1.353			2.462
*TG401*	Z	829	*A.s.*	39	0	0.000	0.000	/	/	2.051
			*A.p.*	45	4	0.027	0.110	−1.764	−2.240	1.697
			*A.d.*	44	1	0.005	0.028	−1.115	−1.803	2.536
			All	128	18	0.712	0.400			2.093
*TG1505*	Z	532	*A.s.*	39	0	0.000	0.000	/	/	1.880
			*A.p.*	43	0	0.000	0.000	/	/	2.444
			*A.d.*	42	0	0.000	0.000	/	/	2.632
			All	124	6	0.508	0.209			2.330

^a^Autosome (A), Chromosome Z (Z)

^b^Length of sequence (bp)

^c^ *A. scirpaceus* (*A.s.*), *A. palustris* (*A.p.*), *A. dumetorum* (*A.d.*)

^d^Number of haploid sequences

^e^Number of segregating sites

^f^Average number of nucleotide differences

^g^Proportion of polymorphic sites

^h^Tajima’s D

^i^Fu and Li’s D

^j^Divergence to outgroup measured as average pairwise divergence, Dxy

Basic population genetic analyses of polymorphism, divergence, recombination, and tests of neutrality based on the allele frequency spectrum were performed with the program DnaSP [32]. Hudson-Kreitman-Aguadé (HKA) tests of positive selection [33] were performed using the HKA program (https://bio.cst.temple.edu/~hey/software/software.htm#HKA). This program compares the ratio of polymorphism to divergence at multiple loci. Loci under positive selection are expected to show lower levels of polymorphism relative to divergence. For each locus, we set the information whether the locus is autosomal or Z-linked, based on which the HKA program accounts for lower effective population size and thus the lower nucleotide variation on the Z chromosome. Genealogical relationships among haplotypes for each locus were reconstructed with Network software [34] using Median joining algorithm.

The data were fit to the isolation-with-migration model (IM) for multiple populations [7] using the program IMa2. The program estimates several demographic parameters based on Markov chain Monte Carlo simulations of genealogies. These parameters include the effective population size for the current and ancestral populations, migration rates between the populations and the population-split times. Because IMa2 assumes no recombination within loci, we determined the longest region without observed recombination for each locus using the program IMgc [35]. This program removes either sites or haplotypes to produce the most information-rich contiguous DNA sequence segment that passes the four-gamete test. Nonrecombinant regions represented 84 % of the length of each locus, on average, and were used as an input for the IMa2 program. For each locus, we provided the information whether it is autosomal or Z-linked, based on which IMa2 program accounts for different effective population sizes between autosomes and the Z chromosome. We ran the program three times with identical starting conditions, with the exception of the random number seed, to assess convergence. To facilitate mixing of the Markov chains, we used Metropolis coupling with 30 chains and a geometric heating model. Upper bounds for the prior distributions of parameter values were set as suggested in IMa2 documentation (10 for population size parameters, 3 for migration rates parameters and 5 for splitting time). All runs began with a burn-in period of 100,000 steps and were allowed to continue for 7–8 million steps. We were able to achieve adequate mixing of the Markov chains as indicated by trend line plots, effective sample size (ESS) values (for all three runs, most ESS values were higher than 20,000 and no ESS value was lower than 50), and very similar parameter estimates in the first half and the second half of the run. Independent runs converged to the same result (e.g., maximum-likelihood estimates and marginal posterior probability distributions for the demographic parameters were essentially the same for all three runs). To perform likelihood-ratio tests of nested models, we combined results of the three independent IMa2 runs (together containing more than 200,000 genealogies) in a single L mode run.

All estimated parameters of the IM model are scaled to the mutation rate. To convert these parameters to biologically meaningful quantities (i.e., *N*_*e*_, effective population size in number of individuals; *m*, migration rate per year; 2 *Nm*, population migration rate; *t*, divergence time in years) we calculated the neutral mutation rate for each locus (*μ*) using divergence to the outgroup. The neutral mutation rate per year was calculated for each locus by using the formula *D* = 2 *μt*_1_ where *D* is the average pairwise divergence, *Dxy*, between the three studied species and the outgroup, and *t*_1_ is the divergence time. We assumed that the divergence time between *A. scirpaceus*/*A. palustris*/*A. dumetorum* and *A. schoenobaenus* is 5.9 million years. This was estimated on the basis of cytochrome b sequence divergence, which is 12.4 % [36], and assuming approximately 2.1 % sequence divergence per million years [37]. The geometric mean of the locus-specific mutation rates was then used for the parameter conversion. A generation time of one year (*Acrocephalus* warblers reproduce only once per year) was assumed to estimate mutation rates per generation.

## Results

### Levels of intraspecific polymorphism and tests of neutrality

**Fig. 2:**
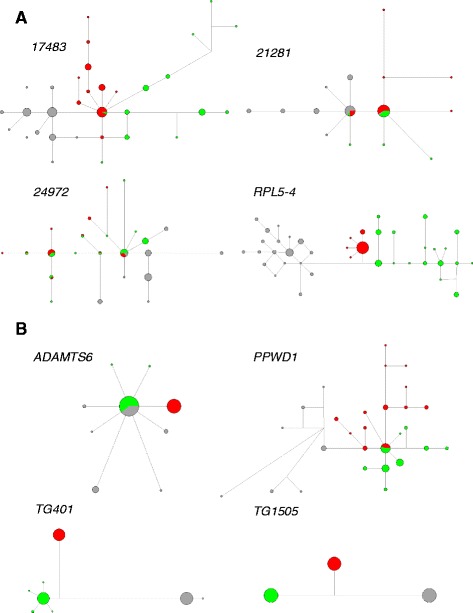
Haplotype networks of four autosomal (**a**) and four Z-linked (**b**) *loci*. Size of the circles are proportional to the number of haplotypes. *A. scirpaceus* is indicated in red, A. palustris in green and A. dumetorum in grey. In the case of *21281 and 24972*, haplotype networks were constructed using only sequences with no recombination witin loci obtained with program IMgc (see Methods)

For each of the eight sequenced loci, we obtained 86 – 128 computationally reconstructed haploid sequences (Table 1). Levels of nucleotide variation were generally high in all three studied species, suggesting a high effective population size of the species. When averaged over all loci, π **=** 0.480 % and θ = 0.575 % in *A. scirpaceus*, π **=** 0.609 % and θ = 0.664 % in *A. palustris*, and π **=** 0.465 % and θ = 0.622 % in *A. dumetorum*. We, however, observed a substantial heterogeneity in the levels of nucleotide variation among loci. Notably, within-species nucleotide variation was zero or very low in three out of four Z-linked loci: *ADAMTS6*, *TG401* and *TG1505*. For *ADAMTS6* and *TG401*, π and θ reached 0 % in *A. scirpaceus* and were also quite low in the remaining two species. For *TG1505,* π and θ reached 0 % even in all three species (Table 1). This can be also seen on haplotype networks showing zero or very low haplotype diversity within the species in the three loci (Fig. 2).

To test whether this heterogeneity in nucleotide variation could be attributed to the action of positive selection at some loci, we performed a multilocus HKA test. The test was applied separately to each species, in each case using a single sequence from *A. schoenobaenus* as the outgroup. The null model was rejected in all three species (Table 2), suggesting that positive selection affects levels of nucleotide variation at one or more loci in all three species. A closer inspection of levels of polymorphism and divergence for individual loci revealed that loci *TG401* in *A. scirpaceus* and *A. palustris*, and *TG1505* in *A. scirpaceus*, *A. palustris* and *A. dumetorum* showed the highest deviations from expected values (Additional file 4). When these two genes were excluded from the HKA test, the null model was not rejected in *A. palustris* and *A. dumetorum*, but was still rejected in *A. scirpaceus* (data not shown). This suggests that a recent selective sweep might have occurred at these two loci independently in multiple species.

**Table 2:** HKA test of positive selection

Species^a^	Sum of deviations^b^	Df^c^	*P*-value^d^	*P*-value^e^
*A.s.*	28.8754	7	**0.00015**	**0.00000**
*A.p.*	17.4805	7	**0.01455**	**0.00310**
*A.d.*	24.4626	7	**0.00094**	**0.00000**

^a^ *A. scirpaceus* (*A.s.*), *A. palustris* (*A.p.*), *A. dumetorum* (*A.d.*)

^b^Counted according to the formula ∑ (observed - expected)^2^/variance)

^c^Degree of freedom

^d^Probability from chi-square distribution (significant values are indicated in bold)

^e^Probability from simulations (no. of simulation 10 000; significant values are indicated in bold)

We also looked for evidence of selection by comparing the distribution of allele frequencies with the expectations under a neutral equilibrium model using Tajima’s D and Fu-Li’s D tests (Table 1). These tests were performed for all studied loci separately in each species. There was a tendency toward negative values of Tajima’s D in all three species (17 of 19 values were negative), which may reflect mild population expansions. Nonetheless, none of the 19 values of Tajima’s D within species were significantly different from the neutral expectation of 0. No significant deviations from neutral expectations were either found in Fu-Li’s D tests. These tests, however, could not be performed for loci with zero within-species nucleotide variation.

### Estimation of divergence times, effective population sizes and rates of interspecific gene flow

**Fig. 3:**
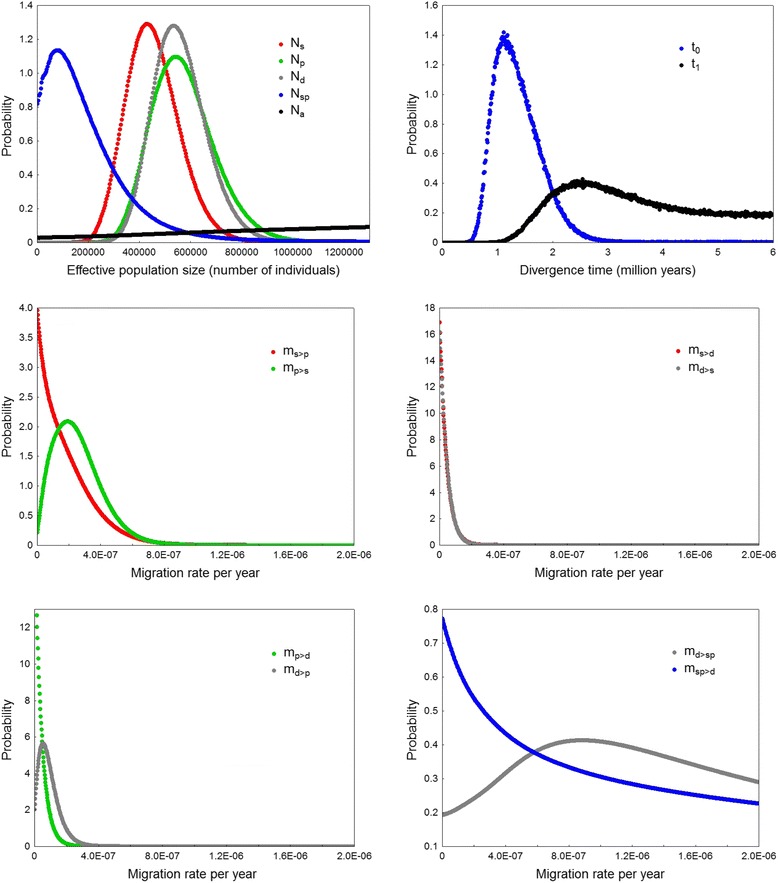
The marginal posterior probability distributions for the demographic parameters of the IM model. Effective population sizes of *A. scirpaceus* (N_s_), *A. palustris* (N_p_), A. *dumetorum* (N_d_), the common ancestor of *A. scirpaceus* and *A. palustris* (N_sp_), and the common ancestor of all three species (Na). Divergence time between *A. scirpaceus* and *A. palustris* (t_0_) and between *A. dumetorum* and the common ancestor of *A. scirpaceus* and *A. palustris* (t_1_). Migration rates per year (m_s>p_ indicates migration from *A. palustris* into *A. scirpaceus*)

Using IM model for multiple populations, we estimated that the time since divergence between *A. scirpaceus* and *A. palustris* is 1.1 Mya and between the common ancestor of these species and *A. dumetorum* 2.5 Mya. The estimated *N*_*e*_ was similar for all three species, 429,802 for *A. scirpaceus*, 541,781 for *A. palustris*, and 535,194 for *A. dumetorum. N*_*e*_ for the common ancestor of *A. scirpaceus* and *A. palustris* was substantially lower, 80,691. *N*_*e*_ for the common ancestor of all three species could not be estimated accurately since the posterior probability distribution for this parameter was very flat (Fig. 3). The estimates of migration rates between *A. scirpaceus* and *A. palustris* were relatively high, although only in one direction (2 *Nm* = 0.238 from *A. scirpaceus* to A*. palustris*, 2 *Nm* < 0.001 in the opposite direction). Migration rates between *A. palustris* and *A. dumetorum* were lower (2 *Nm* = 0.062 from *A. palustris* to *A. dumetorum*, 2 *Nm* < 0.001 in the opposite direction) and no migration was detected between *A. scirpaceus* and *A. dumetorum* (2 *Nm* < 0.001 in both directions). Estimates of migration rates between *A. dumetorum* and the common ancestor of *A. scirpaceus* and *A. palustris* were quite high, though again only in one direction (2 *Nm* = 0.95 from *A. scirpaceus* and *A. palustris* ancestor to *A. dumetorum*, 2 *Nm* < 0.01 in the opposite direction); however, these estimates might not be accurate since posterior probability distributions of these parameter were flat (Fig. 3). Maximum-likelihood estimates (MLE) and 95 % highest posterior density (HPD) intervals of all model parameters are given in Table 3, and the marginal posterior probability distributions are shown in Fig. 3.

**Table 3:** Maximum-likelihood estimates (MLE) and 95 % highest posterior density (HPD) intervals of demographic parameters of isolation with migration model

Parameter	MLE	HPD95Lo^a^	HPD95Hi^b^
N_s_^c^	429802	258540	663640
N_p_^c^	541781	344171	825022
N_d_^c^	535194	360638	772326
N_sp_^c^	80691	0	627412
m_s>p_^d^	1.14E-09	0	5.09E-07
m_p>s_^d^	1.92E-07	7.97E-09	5.43E-07
m_s>d_^d^	1.14E-09	0	1.22E-07
m_d>s_^d^	1.14E-09	0	1.17E-07
m_p>d_^d^	1.14E-09	0	1.24E-07
m_d>p_^d^	5.35E-08	0	2.17E-07
m_d>sp_^d^	8.84E-07	1.81E-07	2.28E-06
m_sp>d_^d^	1.14E-09	0	2.10E-06
2N_s_m_s>p_^e^	0.00081	-	-
2N_s_m_s>d_^e^	0.00039	-	-
2N_p_m_p>s_^e^	0.23800	-	-
2N_p_m_p>d_^e^	0.00054	-	-
2N_d_m_d>s_^e^	0.00044	-	-
2N_d_m_d>p_^e^	0.06182	-	-
2N_d_m_d>sp_^e^	0.94860	-	-
2N_sp_m_sp>d_^e^	0.00750	-	-
t_0_^f^	1116496	668580	2217184
t_1_^f^	2546534	1657289	6583046

^a^Lower 95 % HPD

^b^Higher 95 % HPD

^c^Effective population sizes of *A. scirpaceus* (N_s_), *A. palustris* (N_p_), A. *dumetorum* (N_d_), and the common ancestor of *A. scirpaceus* and *A. palustris* (N_sp_) in numbers of individuals

^d^Migration rates per year (species are marked in the same way as above, e.g., m_s>p_ indicates migration from *A. palustris* into *A. scirpaceus*)

^e^The population migration rate (species are marked in the same way as above, e.g., 2N_s_m_s>p_ indicates population migration rate from *A. palustris* to *A. scirpaceus*)

^f^Divergence time between *A. scirpaceus* and *A. palustris* (t_0_) and between *A. dumetorum* and the common ancestor of *A. scirpaceus* and *A. palustris* (t_1_) in numbers of years

To test whether our estimated model with gene flow fits significantly better the data than models without gene flow between particular species, we performed log-likelihood ratio tests of nested models [38] as implemented in the IMa2 program. We tested four nested models (Table 4) where both migration parameters between (1) *A. scirpaceus* and *A. palustris*, (2) *A. scirpaceus* and *A. dumetorum*, (3) *A. palustris* and *A. dumetorum*, and (4) *A. dumetorum* and the common ancestor of *A. scirpaceus* and *A. palustris* were set to zero. The full model with gene flow was a significantly better fit to the data than all nested models, except the second one (Table 4).

**Table 4:** Log-likelihood ratio tests of nested models

Model^a^	log(P)^b^	2LLR^c^	df^d^	P^e^
m_s>p_ = 0, m_p>s_ = 0	−40.75	106.5	2	**<0.0000**
m_s>d_ = 0, m_d>s_ = 0	12.49	0	2	1.0000
m_p>d_ = 0, m_d>p_ = 0	9.265	6.448	2	**0.0255**
m_p>sp_ = 0, m_sp>d_ = 0	9.333	6.313	2	**0.0273**

^a^Four nested models with zero migration rates between (1) *A. scirpaceus* and *A. palustris*, (2) *A. scirpaceus* and *A. dumetorum*, (3) *A. palustris* and *A. dumetorum*, and (4) *A. dumetorum* and the common ancestor of *A. scirpaceus* and *A. palustris* were compared to the estimated full model

^b^Estimates of the posterior density function under the full model

^c^Log-likelihood ratio statistics calculated as the difference between the highest posterior probability for the full model and the highest posterior probability for the nested model

^d^The degrees of freedom. Models in which migration at least in one direction is equal to 0 have distributions of 2LLR that are a mixture and *χ*2 mixture distribution was thus used to calculate the *P*-value

^e^The probability of achieving the test statistics by chance under the null model (significant values in bold)

We were further interested in whether patterns of interspecific gene flow between *A. scirpaceus* and *A. palustris*, and between *A. palustris* and *A. dumetorum* are similar or not. As IMa2 does not allow to estimate locus-specific migration rates, we compared F_ST_ values, which reflect between-species differentiation and are correlated with levels of gene flow in hybridizing taxa (e.g., [9]). We should, however, note that F_ST_ values might be also affected by the action of positive selection that leads to a reduced within-species nucleotide variation [39]. No statistically significant correlation in F_ST_ between the two species pairs was found (*r* = 0.47, *p* > 0.05), suggesting that the patterns of interspecific gene flow and/or positive selection are in general different for the two species pairs.

## Discussion

Adaptive radiations represent an outstanding system for studying mechanisms of speciation and adaptive divergence. Although divergent ecological selection seems to be an important driver of many adaptive radiations [40], it has been suggested that special genomic properties, such as gene duplications, activation of transposable elements or presence of gene flow among species, can also contribute to high speciation rates and rapid phenotypic changes during adaptive radiations [41].

*Acrocephalus* warblers represent an interesting model system for studying mechanisms of radiation in birds, although their mainly continental radiation was relatively slow compared to the well-known cases of adaptive radiations on islands, such as Darwin finches [13, 17]. Our estimates of divergence times are approximately 1.1 Mya for the sister species *A. scirpaceus* and *A. palustris*, and 2.5 Mya for their common ancestor and *A. dumetorum*. Despite this relatively deep divergence, many currently existing *Acrocephalus* species still hybridize in nature (Additional file 1). This hybridization might in principle lead to gene flow among the species if F_1_ hybrids are fertile. However, since very little is known about the degree of postzygotic isolation in this avian group, it is hard to predict whether gene flow could occur among *Acrocephalus* species and how strong it might be.

Here we provided the first evidence that interspecific gene flow occurred during *Acrocephalus* radiation and not only between sister species. We found evidence of gene flow between *A. scirpaceus* and *A. palustris*, which are the sister species with somewhat different ecological requirements, and between *A. palustris* and *A. dumetorum*, which have similar ecological requirements and often co-occur at the same sites where their ranges overlap. Significant gene flow was detected also between *A. dumetorum* and the ancestor of *A. scirpaceus* and *A. palustris*. No gene flow was, however, detected between *A. scirpaceus* and *A. dumetorum*. Although the isolation-with-migration model estimates not only contemporary gene flow, but average gene flow among populations since the time of divergence [7], it is noteworthy that hybridization currently occurs between both pairs of species for which gene flow has been detected, but not between *A. scirpaceus* and *A. palustris* for which no clear cases of interspecific hybridization are known.

Interestingly, the estimated levels of gene flow were in all cases asymmetric. Gene flow occurred from *A. scirpaceus* to *A. palustris* (2 *Nm* = 0.238), from *A. palustris* to *A. dumetorum* (2 *Nm* = 0.062), and from the *A. scirpaceus*/*A. palustris* ancestor to *A. dumetorum* (2 *Nm* = 0.95). Zero gene flow was detected in the opposite directions. Such unidirectional gene flow is expected when a population of one species expands to the area already occupied by a related species and interbreeding is not prevented between the two species. Introgression of neutral alleles then occurs almost exclusively from the local into the invading species [42]. Unfortunately, history of breeding range changes are not well known for the studied species, but historically recent breeding range expansion of *A. dumetorum* to eastern and northern Europe where it encountered *A. palustris* [20] is consistent with this scenario.

An important issue in speciation research is to understand evolutionary forces that are responsible for the establishment of reproductive barriers between the species. Here we tested whether gene flow is higher between genetically more similar, but ecologically somewhat different, *A. scirpaceus* and *A. palustris*, or between ecologically more similar, but genetically more distant, *A. palustris* and *A. dumetorum*. Our results show that gene flow is more than three times higher between genetically more similar *A. scirpaceus* and *A. palustris* than between *A. palustris* and *A. dumetorum*, although the latter two species have higher chance to meet in the breeding sites and very likely more often hybridize. Lower levels of gene flow between *A. palustris* and *A. dumetorum* might be caused by historically more recent contact between the species [23], but might also suggest that divergent ecological selection is not the major cause of reproductive isolation and that speciation in *Acrocephalus* warblers is more likely driven by gradual accumulation of postzygotic barriers, such as hybrid sterility. Hybrid sterility have been thought to evolve quite slowly in birds compared to other taxonomic groups; however, recent findings suggest that at least in some bird species it can evolve very rapidly [43]. It is, however, also possible that very different migratory behaviors of *A. dumetorum*, wintering in India, and *A. palustris*, that winters similarly as *A. scirpaceus* in Africa (although in different regions), might have contributed to their stronger reproductive isolation. Indeed, studies in Swainson’s Thrush (*Catharus ustulatus*) show that different seasonal migratory behavior of its two subspecies can be a source of selection against hybrids [44]. Analysis of more species with different genetic and ecological similarity would be needed to conclude which selective forces drive radiation of *Acrocephalus* warblers.

Although the patterns of between species differentiation were in general different for the two species pairs, we detected signatures of apparently independent instances of positive selection at the same two Z-linked loci in multiple species. Selective sweeps reducing the within-species nucleotide variation occurred independently in *A. scirpaceus* and *A. palustris* at *TG401* locus and even in all three species at *TG1505* locus. Although we do not know the physical location of the two loci in the *Acrocephalus* genome, the two loci lie in different parts of the Z chromosome in the Zebra Finch genome. Given the very conserved karyotypes in birds [45], it is likely that the two loci are also unlinked in *Acrocephalus* genome and that selection has led to reduced nucleotide variation independently at the two loci. Similar examples of reduced nucleotide diversity at the same loci in multiple species have been observed for example in a genomic scan of islands of divergence in two hybridizing *Ficedula* flycatchers [10] or between two closely related and hybridizing plants, *Silene latifolia* and *S. dioica* [46]. Such independent instances of positive selection in multiple species suggest that the same (or closely linked) loci might have been subject to parallel selection in two or even all three *Acrocephalus* species. This might happen for example in genes involved in intragenomic conflicts, such as meiotic drive, which is expected to be especially common on the sex chromosomes. Whether divergence of these genes could contribute to *Acrocephalus* speciation, however, still remains to be explored. In addition, it should be tested whether similar patterns of adaptive evolution in the three *Acrocephalus* species could not result from linked selection in low-recombinant regions rather than parallel adaptive evolution [47].

## Conclusions

Our study provides the first evidence that interspecific gene flow occurred during *Acrocephalus* radiation and not only between sister species. Interspecific gene flow could thus play an important role in shaping genetic variation in individual species and possibly accelerate adaptive evolution and speciation rates in this group. Interestingly, the gene flow was in all cases asymmetric, suggesting that it might have occurred mainly during range expansions of individual species into areas of closely related and not completely reproductively isolated species. Interspecific competition leading to ecological character displacement and habitat segregation [48] and/or reinforcement of prezygotic isolation [49] could then strengthen reproductive barriers between the species. However, in contrast to Darwin finches [13], our results showed that gene flow was higher between genetically more closely related but ecologically more divergent species suggesting that gradual accumulation of genetic incompatibilities causing intrinsic isolation rather than divergent ecological selection are more efficient in restricting interspecific gene flow in this avian group. Thus in contrast to Darwin finches [13], the ecological differentiation of *Acrocephalus* species likely arose in later stages of speciation. Data on more than three *Acrocephalus* species would be, however, needed to generalize this conclusion to the whole *Acrocephalus* radiation. In addition, genome-wide data across autosomes and the whole Z chromosome would be needed to understand mechanisms responsible for the parallel adaptive evolution at the same loci in multiple species and its possible role in *Acrocephalus* speciation. Nevertheless, occurrence of interspecific hybridization among many *Acrocephalus* species suggests that interspecific gene flow could be common across the whole radiation.

## Additional files

Additional file 1: Published cases of interspecific mating (a) and interspecific hybrids (b) among *Acrocephalus* species. (DOC 76 kb) Additional file 2: List of analyzed samples including information of sex of each individual and geographic coordinates of the sites, where individual birds were captured. (DOC 103 kb) Additional file 3: PCR primers and length of PCR products. (DOC 38 kb) Additional file 4: Observed and expected levels of polymorphism and divergence in HKA test of positive selection. (DOC 63 kb)
