# Supplementary material for: Patterns of gene flow and selection across multiple species of Acrocephalus warblers: footprints of parallel selection on the Z chromosome
Source: BMC Evol Biol. 2016 Jun 16;16:130. doi: 10.1186/s12862-016-0692-2 (PMC4910229; doi:10.1186/s12862-016-0692-2)
Supplement: Additional file 1: — Published cases of interspecific mating (a) and interspecific hybrids (b) among Acrocephalus species. (DOC 76 kb) [file 12862_2016_692_MOESM1_ESM.doc]

**Additional file 1.**

Published cases of interspecific mating (a) and interspecific hybrids (b) among *Acrocephalus* species (the three study species in bold).

**a) interspecific mating**

| **Species pair** | **N** | **Female** | **Rarer species** | **Country** | **Reference** |
| --- | --- | --- | --- | --- | --- |
| *agricola × scirpaceus* | 1 | *agricola* | *agricola* | Kazakhstan | A. Pukas in Panov 1989 |
| ***dumetorum × palustris*** | 1 | *palustris* | *dumetorum* | Netherlands | Poot et al. 1999; van Loon & Keijl 2001 |
|  | 1 | *palustris* | *dumetorum* | Norway | Dyresen 1999 |
|  | >7 | *palustris* | *palustris* | Finland | Koskimies 1980, 1984, 1991 |
|  | 1 | *dumetorum* | *palustris* | Finland | Koskimies 1991 |
|  | 1 | *dumetorum × palustris* | *palustris* | Finland | Koskimies 1984 |
| ***palustris × scirpaceus*** | 1 | *scirpaceus* | *-* | Sweden | Lundwall & Persson 1987 |
|  | 2 | *scirpaceus* | *scirpaceus* | Germany | Walter 2010 |
|  | 1 | *scirpaceus* | both common | Germany | Diesselhorst 1948 |
|  | 1 | *scirpaceus* | *palustris* | UK | M. G. Kelsey in Cramp 1992 (p. 201) |
|  | 1 | *palustris* | *scirpaceus* | Belgium | Lemaire 1977 |
|  | 3 | *palustris* | both common | Lithuania | Pukas 1989 |
|  | 1 | *-* | - | Germany | K. Kräuter in Schulze-Hagen K. 1991 (p. 425) |
| ***scirpaceus × dumetorum*** | 0 |  |  |  |  |

**b) interspecific hybrids**

| **Species pair** | **N** | **Mother** | **Evidence** | **Country** | **Reference** |
| --- | --- | --- | --- | --- | --- |
| *arundinaceus × stentoreus* | 8 | *-* | biometry; song | Kazakhstan | A. Pukas in Panov 1989 |
|  | 4 | *stentoreus* (3), *arundinaceus* (1) | biometry; mtDNA, microsat; song | Kazakhstan | Hansson *et al.* 2003 |
| *arundinaceus × scirpaceus* | 1 | *arundinaceus* | biometry; mtDNA, microsat; song | Germany | Beier *et al.* 1997 |
|  | 1 | *arundinaceus* | biometry; microsatellites | Belgium | Hansson *et al.* 2004 |
|  | 1 | *arundinaceus* | biometry; cytb, COI, microsat | Romania | Ion *et al.* 2012 |
| ***dumetorum × palustris*** | 1 | *dumetorum* | biometry; mtDNA cytb; song | Finland | Lindholm *et al.* 2007 |
|  | 2 | *-* | biometry | Finland | Finnish ringing scheme quoted by Lindholm *et al.* 2007 |
|  | several | *-* | biometry; song | Finland | Koskimies 1991 |
|  | 1 | *-* | biometry; song | Finland | Kosonen 1983 |
|  | 1 | *palustris* | biometry; mtDNA cytb | Kenya | A Rocha Kenya 2010 |
|  | 4 | *-* | biometry | Sweden | Lundwall & Persson 1987 |
| ***palustris × scirpaceus*** | 1 | *-* | biometry | Finland | Haataja & Kaarto 1984 |
|  | 3 | *-* | biometry | Lithuania | Pukas 1989 |
|  | 2 | *-* | song | Germany | Boxberger 1949 |
|  | 8 | *-* | biometry | France | P. Collette in Lemaire 1977 |
|  | 1 | *-* | biometry | Belgium | Lemaire 1977 |
|  | 1 | *-* | biometry | Sweden | Lundwall & Persson 1987 |
|  | 2 | *scirpaceus* | biometry; parents caught | Sweden | Lundwall & Persson 1987 |
|  | 1 | *scirpaceus* | biometry; mtDNA cytb; song | Norway | Otterbeck *et al.* 2013 |
| ***scirpaceus × dumetorum*** | 1 | *-* | song | Sweden | Hagström 1984 |
| *scirpaceus × schoenobaenus* | 1 | *-* | biometry, coloration | Netherlands | Van Eerde 1999 |
|  | 4 | *-* | coloration | Finland | Sharrock 1985 |
|  | 1 | *-* | coloration | UK | Wormwell 2003 |
| *schoenobaenus × palustris* | 1 | *palustris* | mtDNA COI, microsat; coloration; song | Norway | Lifjeld *et al.* 2010 |

**Full references**

A. Pukas in Panov EN (1989). Gibridizatsiya i etologicheskaya izolyatsiya u ptits. Nauka, Moskva.

A Rocha Kenya (2010). Putative Blyth's reed warbler turns out different; Ngulia update - probable first record for Africa: Blyth's reed warbler. http://arochakenya.wildlifedirect.org/tag/blyths-reed-warbler/

Beier J, Leisler B, Wink M (1997). Ein Drossel- × Teichrohrsänger-Hybride *Acrocephalus arundinaceus × A. scirpaceus* und der Nachweis seiner Elternschaft. *J Ornithol* **138**: 51-60.

Boxberger L von (1949). Zur Frage der Bastardierung von Teich- und Sumpfrohrsänger. *Ornithol Ber* **2**: 48.

Cramp J (ed.) (1992). *Birds of the Western Palaearctic*. Vol. 6. Oxford University Press, Oxford

Diesselhorst G (1948). Eine Mischbrut Sumpfrohrsänger × Teichrohrsänger. *Ornithol Berichte* **1**: 239.

Dyresen A (1999). Nattsangerregistreringer i Østvold 1996. *Natur i Østvold* **18**: 65-76.

Hansson B, Gavrilov E, Gavrilov A (2003). Hybridization between great reed warblers *Acrocephalus arundinaceus* and clamorous reed warblers *A. stentoreus*: morphological and molecular evidence. *Avian Science* **3**: 145-151.

Hansson B, Roggeman W, De Smet G (2004). Molecular evidence of a reed warbler × great reed warbler (*Acrocephalus scirpaceus × A. arundinaceus*) in Belgium. *J Ornithol* **145**: 159-160.

Haataja K, Kaarto I (1984). Mahdollinen ryti- ja luhtakerttusen risteymä (*Acrocephalus scirpaceus* × *palustris*) Oulussa. *Aureola* **9**: 46–49.

Hagström B (1984). Om en hybrid mellan rör- och busksångare. *Vingspegeln* **3**: 167.

Ion C, Bolboaca L, Ciorpac M, Stefan A, Gorgan DL (2012). A great reed warbler × reed warbler hybrid (*Acrocephalus arundinaceus* × *Acrocephalus scirpaceus*) in northeastern Romania. *J Ornithol* **153**: 975-978.

Koskimies P (1980). Breeding biology of Blyth's reed warbler *Acrocephalus dumetorum* in SE Finland. *Ornis Fenn* **57**: 26-32.

Koskimies P (1984). Polygyny in Blyth's reed warbler *Acrocephalus dumetorum*. *Ann Zool Fennici* **21**: 239-242.

Koskimies P (1991). *Acrocephalus dumetorum* Blyth 1849 - Buschrohrsänger In: Glutz von Blotzheim UN & Bauer KM 1991 (eds): *Handbuch der Vögel Mitteleuropas*. Vol. 12/I. AULA-Verlag, Wiesbaden: 352-376

Kosonen L (1983). Todennäköinen vita- ja luhtakerttusen risteymä *Acrocephalus dumetorum* × *palustris* Tampereella. *Lintuviesti* **8**: 210-213.

Lemaire F (1977). Mixed song, interspecific competition and hybridisation in the reed and marsh warblers (*Acrocephalus scirpaceus* and *palustris*). *Behaviour* **63**: 215-239.

Lifjeld JT, Marthinsen G, Myklebust M, Dawson DA, Johnsen A (2010). A wild marsh warbler × sedge warbler hybrid (*Acrocephalus palustris × A. schoenobaenus*) in Norway documented with molecular markers. *J Ornithol* **151**: 513-517.

Lindholm A, Bensch S, Dowsett-Lemaire F (2007). Hybrid Marsh × Blyth's Reed Warbler with mixed song in Finland in June 2003. *Dutch Birding* **29**: 223-231.

Loon AJ, Keijl GO (2001). Blyth's reed warbler at Nieuwegein in June-July 1998. *Dutch Birding* **23**: 83–85.

Lundwall U, Persson C (1987). Bestämningsproblem III: *Acrocephalus*-hybrider. *Fågelstudier* **3**: 85-86.

Otterbeck A, Dale S, Lindén A, Marthinsen G (2013). A male Reed Warbler and Marsh Warbler hybrid (*Acrocephalus scirpaceus × A. palustris*) in Norway documented with molecular methods. *Ornis Norvegica* **36**: 6-13.

Poot M, Engelen F, van der Winden J (1999). Een gemengd broedgeval van Struikrietzanger *Acrocephalus dumetorum* en Bosrietzanger *A. palustris* bij Utrecht in voorjaar 1998. *Limosa* **72**: 151-157.

Pukas A (1989). Sluchai obrazovaniya smeshannykh par trostnikovoy (*Acrocephalus scirpaceus*) i bolotnoy (*Acrocephalus palustris*) kamyshevok (Passeriformes, Sylviidae). *Zool zhurnal* **64**: 153-157.

Schulze-Hagen K (1991). *Acrocephalus palustris* - Sumpfrohrsänger. In: Glutz UN, Bauer KM (eds): *Handbuch der Vögel Mitteleuropas*. AULA-Verlag, Wiesbaden: 377-433.

Sharrock JTR (1985). Mystery Photographs 105. *Brit Birds* **78**: 434–437.

Van Eerde KA (1999). Hybride Rietzanger × Kleine Karekiet te Makkum in augustus 1997. *Dutch Birding* **21**: 34-37.

Walter D (2010). Brutbiologie, Phänologie und Bestandsentwicklung einer voralpinen Population des Sumpfrohrsängers *Acrocephalus palustris* im Allgäu (Bayern/Deutschland). *Ornithol Anz* **49**: 103-148.

Wormwell C (2003). The strange warbler at the Lhen 15 & 16/06/03. http://www.homepages.mcb.net/wormwell/strange_warbler_at_the_lhen_15.htm
