# Supplementary material for: Patterns of gene flow and selection across multiple species of Acrocephalus warblers: footprints of parallel selection on the Z chromosome
Source: BMC Evol Biol. 2016 Jun 16;16:130. doi: 10.1186/s12862-016-0692-2 (PMC4910229; doi:10.1186/s12862-016-0692-2)
Supplement: Additional file 2: — List of analyzed samples including information of sex of each individual and geographic coordinates of the sites, where individual birds were captured. (DOC 103 kb) [file 12862_2016_692_MOESM2_ESM.doc]

**Additional file 2.**

List of analyzed samples including information of sex of each individual and geographic coordinates of the sites, where individual birds were captured.

| ID | Species^1^ | Date^2^ | Site | Sex^3^ | GPS position | |
| --- | --- | --- | --- | --- | --- | --- |
| R14 | *A. s.* | 23/6/2010 | Rauvola, Kaarina | M | 60°40.601’N | 22°28.520’E |
| R15 | *A. s.* | 23/6/2010 | Rauvola, Kaarina | F | 60°40.601’N | 22°28.520’E |
| R16 | *A. s.* | 23/6/2010 | Rauvola, Kaarina | M | 60°40.601’N | 22°28.520’E |
| R17 | *A. s.* | 23/6/2010 | Rauvola, Kaarina | M | 60°40.601’N | 22°28.520’E |
| R18 | *A. p.* | 24/6/2010 | Vintala, Lieto | M | 60°55.024’N | 22°45.800’E |
| R19 | *A. s.* | 24/6/2010 | Aarlahti, Mynämäki | M | 60°63.032’N | 21°81.482’E |
| R20 | *A. s.* | 25/6/2010 | Raisio | F | 60°47.411’N | 22°12.399’E |
| R21 | *A. p.* | 25/6/2010 | Raisio | M | 60°47.411’N | 22°12.399’E |
| R22 | *A. p.* | 25/6/2010 | Raisio | M | 60°47.411’N | 22°12.399’E |
| R23 | *A. s.* | 25/6/2010 | Raisio | M | 60°47.411’N | 22°12.399’E |
| R24 | *A. p.* | 25/6/2010 | Raisio | M | 60°47.411’N | 22°12.399’E |
| R25 | *A. d.* | 26/6/2010 | Tarvasjoki | M | 60°57.402’N | 22°75.211’E |
| R26 | *A. d.* | 26/6/2010 | Lieto | M | 60°46.845’N | 22°35.348’E |
| R27 | *A. s.* | 26/6/2010 | Viurila, Salo | F | 60°37.597’N | 23°06.829’E |
| R28 | *A. s.* | 26/6/2010 | Viurila, Salo | M | 60°37.597’N | 23°06.829’E |
| R29 | *A. d.* | 27/6/2010 | Iso-Hiisi, Pertteli | M | 60°47.118’N | 23°33.236’E |
| R30 | *A. p.* | 27/6/2010 | Salo | M | 60°40.581’N | 23°16.106’E |
| R31 | *A. s.* | 27/6/2010 | Salo | M | 60°36.772’N | 23°09.071’E |
| R32 | *A. p.* | 27/6/2010 | Pullola, Salo | M | 60°32.288’N | 23°21.950’E |
| R33 | *A. d.* | 28/6/2010 | Kisko | M | 60°26.370’N | 23°44.241’E |
| R34 | *A. p.* | 28/6/2010 | Svartå, Karjaa | M | 60°14.068’N | 23°83.176’E |
| R35 | *A. p.* | 28/6/2010 | Svartå, Karjaa | M | 60°14.068’N | 23°83.176’E |
| R36 | *A. p.* | 29/6/2010 | Iso-Huopalahti, Espoo | M | 60°21.104’N | 24°84.445’E |
| R37 | *A. d.* | 29/6/2010 | Iso-Huopalahti, Espoo | M | 60°21.104’N | 24°84.445’E |
| R38 | *A. p.* | 29/6/2010 | Iso-Huopalahti, Espoo | F | 60°21.104’N | 24°84.445’E |
| R39 | *A. d.* | 29/6/2010 | Iso-Huopalahti, Espoo | M | 60°21.104’N | 24°84.445’E |
| R40 | *A. d.* | 29/6/2010 | Iso-Huopalahti, Espoo | M | 60°21.104’N | 24°84.445’E |
| R41 | *A. d.* | 29/6/2010 | Iso-Huopalahti, Espoo | F | 60°21.104’N | 24°84.445’E |
| R42 | *A. d.* | 29/6/2010 | Iso-Huopalahti, Espoo | F | 60°21.104’N | 24°84.445’E |
| R43 | *A. p.* | 29/6/2010 | Iso-Huopalahti, Espoo | M | 60°21.104’N | 24°84.445’E |
| R44 | *A. p.* | 29/6/2010 | Iso-Huopalahti, Espoo | M | 60°21.104’N | 24°84.445’E |
| R45 | *A. s.* | 29/6/2010 | Iso-Huopalahti, Espoo | M | 60°20.965’N | 24°84.865’E |
| R46 | *A. s.* | 29/6/2010 | Iso-Huopalahti, Espoo | M | 60°20.965’N | 24°84.865’E |
| R47 | *A. s.* | 29/6/2010 | Iso-Huopalahti, Espoo | M | 60°20.965’N | 24°84.865’E |
| R48 | *A. s.* | 29/6/2010 | Iso-Huopalahti, Espoo | M | 60°20.965’N | 24°84.865’E |
| R49 | *A. s.* | 30/6/2010 | Laajalahti, Espoo | M | 60°19.753’N | 24°81.636’E |
| R50 | *A. s.* | 30/6/2010 | Espoonlahti, Espoo | F | 60°14.281’N | 24°69.747’E |
| R51 | *A. p.* | 30/6/2010 | Espoonlahti, Espoo | M | 60°14.281’N | 24°69.747’E |
| R52 | *A. s.* | 30/6/2010 | Espoonlahti, Espoo | M | 60°14.281’N | 24°69.747’E |
| R53 | *A. p.* | 1/7/2010 | Bemböle, Espoo | F | 60°22.308’N | 24°67.685’E |
| R54 | *A. p.* | 1/7/2010 | Bemböle, Espoo | M | 60°22.308’N | 24°67.685’E |
| R55 | *A. d.* | 1/7/2010 | Bemböle, Espoo | M | 60°22.308’N | 24°67.685’E |
| R56 | *A. p.* | 1/7/2010 | Bemböle, Espoo | M | 60°22.308’N | 24°67.685’E |
| R57 | *A. p.* | 1/7/2010 | Bemböle, Espoo | F | 60°22.308’N | 24°67.685’E |
| R58 | *A. p.* | 2/7/2010 | Vantaa | F | 60°27.727’N | 24°94.613’E |
| R59 | *A. p.* | 2/7/2010 | Vantaa | M | 60°27.727’N | 24°94.613’E |
| R60 | *A. p.* | 2/7/2010 | Vantaa | M | 60°27.727’N | 24°94.613’E |
| R61 | *A. s.* | 2/7/2010 | Kurttila, Espoo | M | 60°17.310’N | 24°58.228’E |
| R62 | *A. s.* | 2/7/2010 | Kurttila, Espoo | M | 60°17.310’N | 24°58.228’E |
| R63 | *A. s.* | 2/7/2010 | Kurttila, Espoo | F | 60°17.310’N | 24°58.228’E |
| R64 | *A. s.* | 2/7/2010 | Kurttila, Espoo | M | 60°17.310’N | 24°58.228’E |
| R65 | *A. p.* | 3/7/2010 | Toikansuo, Lappeeranta | F | 61°03.182’N | 28°19.500’E |
| R66 | *A. p.* | 3/7/2010 | Toikansuo, Lappeeranta | M | 61°03.182’N | 28°19.500’E |
| R67 | *A. d.* | 3/7/2010 | Korvenkylä, Joutseno | M | 61°15.172’N | 28°64.170’E |
| R68 | *A. d.* | 3/7/2010 | Korvenkylä, Joutseno | M | 61°15.172’N | 28°64.170’E |
| R69 | *A. d.* | 3/7/2010 | Korvenkylä, Joutseno | F | 61°15.172’N | 28°64.170’E |
| R70 | *A. d.* | 3/7/2010 | Korvenkylä, Joutseno | M | 61°15.172’N | 28°64.170’E |
| R71 | *A. d.* | 3/7/2010 | Korvenkylä, Joutseno | M | 61°15.172’N | 28°64.170’E |
| R72 | *A. d.* | 4/7/2010 | Korvenkylä, Joutseno | M | 61°15.172’N | 28°64.170’E |
| R73 | *A. d.* | 4/7/2010 | Korvenkylä, Joutseno | M | 61°15.172’N | 28°64.170’E |
| R74 | *A. d.* | 4/7/2010 | Korvenkylä, Joutseno | M | 61°16.489’N | 28°68.316’E |
| R75 | *A. d.* | 4/7/2010 | Korvenkylä, Joutseno | F | 61°16.489’N | 28°68.316’E |
| R76 | *A. d.* | 4/7/2010 | Korvenkylä, Joutseno | M | 61°16.489’N | 28°68.316’E |
| R77 | *A. p.* | 4/7/2010 | Korvenkylä, Joutseno | M | 61°16.489’N | 28°68.316’E |
| R78 | *A. p.* | 4/7/2010 | Korvenkylä, Joutseno | F | 61°16.489’N | 28°68.316’E |
| R79 | *A. d.* | 4/7/2010 | Korvenkylä, Joutseno | F | 61°16.489’N | 28°68.316’E |
| R80 | *A. p.* | 4/7/2010 | Korvenkylä, Joutseno | M | 61°16.489’N | 28°68.316’E |
| R81 | *A. p.* | 4/7/2010 | Korvenkylä, Joutseno | F | 61°16.489’N | 28°68.316’E |
| R82 | *A. d.* | 4/7/2010 | Korvenkylä, Joutseno | M | 61°16.489’N | 28°68.316’E |
| R83 | *A. d.* | 5/7/2010 | Toikansuo, Lappeenranta | F | 61°03.926’N | 28°19.017’E |
| R84 | *A. d.* | 5/7/2010 | Toikansuo, Lappeenranta | M | 61°03.926’N | 28°19.017’E |
| R85 | *A. d.* | 5/7/2010 | Toikansuo, Lappeenranta | M | 61°03.926’N | 28°19.017’E |
| R86 | *A. s.* | 7/7/2010 | Pernåviken, Pernå | M | 60°48.076’N | 25°92.955’E |

^1^ *A. scirpaceus* (*A.s.*), *A. palustris* (*A.p.*), *A. dumetorum* (*A.d.*).

^2^ Date of capturing.

^3^ Male (M), Female (F).
