# Supplementary material for: Patterns of gene flow and selection across multiple species of Acrocephalus warblers: footprints of parallel selection on the Z chromosome
Source: BMC Evol Biol. 2016 Jun 16;16:130. doi: 10.1186/s12862-016-0692-2 (PMC4910229; doi:10.1186/s12862-016-0692-2)
Supplement: Additional file 3: — PCR primers and length of PCR products. (DOC 38 kb) [file 12862_2016_692_MOESM3_ESM.doc]

**Additional file 3.** PCR primers and length of PCR products.

| **Locus** | **Primer Direction** | **Primer sequence** | **Length^1^** | **Reference** |
| --- | --- | --- | --- | --- |
| *17483* | F | GAAATGTGGTCTGAACAGTC | 650 | Backström *et al.* 2008 |
|  | R | TTGCTCTTGGCACGATATGC |  |  |
| *21281* | F | GACCAAGACAACTTCCTGCC | 950 | Backström *et al.* 2008 |
|  | R | GTAGATTTCGACACCTCCAG |  |  |
| *24972* | F | CGTTCCACTAATATTTTCCG | 1000 | Backström *et al.* 2008 |
|  | R | GCTTCATCAGTGACTATGAC |  |  |
| *RPL5-4* | F | GTTGGCCTGACCAATTACGC | 750 | Borge *et al.* 2005 |
|  | R | CTTCAACTTGGCCTTCATAGATCTT |  |  |
| *ADAMTS6* | F | GGAGAGAATGGATTTCTGCC | 700 | Backström *et al.* 2006 |
|  | R | TGATTCCAGTCTAGGAAACG |  |  |
| *PPWD1* | F | AACTGTGGAAAACTTCTGTG | 850 | Backström *et al.* 2006 |
|  | R | TCATCTTCAAATTCTCCTCC |  |  |
| *TG401* | F | TTTTCTCCAGCATTGCACTT | 1150 | This study |
|  | R | TTTTCTTCTTTCTCCAGATAGCG |  |  |
| *TG1505* | F | CCCAACACCCGTGCTAACTA | 750 | This study |
|  | R | AGACCTCCTTGAACATCCCA |  |  |

^1^ Length of PCR product

**Full references**

Borge T, Webster MT, Andersson G and Saetre GP (2005). Contrasting patterns of polymorphism and divergence on the Z chromosome and autosomes in two *Ficedula* flycatcher species. *Genetics* **171**: 1861-1873.

Backström N, Brandström M, Gustafsson L, Qvarnström A, Cheng H, Ellegren H (2006). Genetic mapping in a natural population of collared flycatchers (*Ficedula albicollis*): conserved synteny but gene order rearrangements on the avian Z chromosome. *Genetics* **174**: 377–386.

Backström N, Fagerberg S, Ellegren H (2008). Genomics of natural bird populations: a gene-based set of reference markers evenly spread across the avian genome. *Mol Ecol* **17**: 964–980.
