# Supplementary material for: Patterns of gene flow and selection across multiple species of Acrocephalus warblers: footprints of parallel selection on the Z chromosome
Source: BMC Evol Biol. 2016 Jun 16;16:130. doi: 10.1186/s12862-016-0692-2 (PMC4910229; doi:10.1186/s12862-016-0692-2)
Supplement: Additional file 4: — Observed and expected levels of polymorphism and divergence in HKA test of positive selection. (DOC 63 kb) [file 12862_2016_692_MOESM4_ESM.doc]

**Additional file 4.** Observed and expected levels of polymorphism and divergence in HKA test of positive selection.

|  |  | Polymorphic sites within species | | | |  | Divergence to outgroup ^2^ | | | |
| --- | --- | --- | --- | --- | --- | --- | --- | --- | --- | --- |
| Locus | Species^1^ | Obs. | Exp. | Var. | Dev. ^3^ |  | Obs. | Exp. | Var. | Dev. ^3^ |
| *17483* | *A.s.* | 9 | 12.03 | 24.44 | 0.376 |  | 15.84 | 12.81 | 20.46 | 0.449 |
|  | *A.p.* | 13 | 15.34 | 38.75 | 0.141 |  | 17.66 | 15.32 | 29.83 | 0.184 |
|  | *A.d.* | 10 | 13.72 | 29.23 | 0.473 |  | 17.40 | 13.68 | 23.23 | 0.595 |
| *21281* | *A.s.* | 29 | 18.10 | 52.90 | 2.245 |  | 10.64 | 21.54 | 43.18 | 2.751 |
|  | *A.p.* | 24 | 15.63 | 46.58 | 1.503 |  | 9.35 | 17.72 | 37.13 | 1.885 |
|  | *A.d.* | 22 | 16.14 | 40.00 | 0.860 |  | 11.13 | 17.00 | 31.75 | 1.083 |
| *24972* | *A.s.* | 29 | 20.70 | 69.94 | 0.985 |  | 17.38 | 25.67 | 56.40 | 1.221 |
|  | *A.p.* | 35 | 26.20 | 89.10 | 0.870 |  | 16.26 | 25.07 | 63.94 | 1.212 |
|  | *A.d.* | 30 | 22.60 | 69.40 | 0.790 |  | 16.40 | 23.80 | 52.72 | 1.040 |
| *RPL5-4* | *A.s.* | 4 | 9.13 | 16.76 | 1.567 |  | 15.18 | 10.06 | 14.78 | 1.778 |
|  | *A.p.* | 15 | 16.86 | 39.89 | 0.087 |  | 17.00 | 15.14 | 29.31 | 0.118 |
|  | *A.d.* | 12 | 13.92 | 29.88 | 0.123 |  | 15.79 | 13.88 | 23.71 | 0.155 |
| *ADAMTS6* | *A.s.* | 0 | 2.07 | 2.47 | 1.727 |  | 5.00 | 2.93 | 3.18 | 1.342 |
|  | *A.p.* | 2 | 2.80 | 3.47 | 0.183 |  | 4.05 | 3.25 | 3.67 | 0.173 |
|  | *A.d.* | 7 | 5.04 | 7.27 | 0.526 |  | 4.57 | 6.53 | 7.90 | 0.484 |
| *PPWD1* | *A.s.* | 14 | 11.64 | 24.72 | 0.226 |  | 14.27 | 16.63 | 24.73 | 0.226 |
|  | *A.p.* | 9 | 10.75 | 20.56 | 0.149 |  | 14.11 | 12.36 | 18.40 | 0.167 |
|  | *A.d.* | 15 | 9.83 | 25.53 | 1.048 |  | 12.46 | 17.63 | 27.66 | 0.967 |
| *TG401* | *A.s.* | 0 | 7.14 | 11.75 | **4.335** |  | 17.00 | 9.86 | 12.71 | **4.008** |
|  | *A.p.* | 4 | 8.40 | 14.40 | 1.347 |  | 14.07 | 9.66 | 13.36 | 1.452 |
|  | *A.d.* | 1 | 9.66 | 17.66 | **4.247** |  | 21.02 | 12.36 | 17.29 | **4.336** |
| *TG1505* | *A.s.* | 0 | 4.20 | 5.80 | **3.042** |  | 10.00 | 5.80 | 6.79 | 2.597 |
|  | *A.p.* | 0 | 6.01 | 9.14 | **3.954** |  | 13.00 | 6.99 | 8.92 | **4.054** |
|  | *A.d.* | 0 | 6.10 | 9.36 | **3.978** |  | 14.00 | 7.90 | 9.91 | **3.759** |

| ^1^ *A. scirpaceus* (*A.s.*), *A. palustris* (*A.p.*), *A. dumetorum* (*A.d.*). |
| --- |
| ^2^ *A. schoenobaenus* was used as an outgroup |
| ^3^ Deviation for each observation (i.e. (observed - expected)2/variance)). Values higher than 3 are indicated in bold. |
